# Differences in health care spending and utilization among older frail adults in high‐income countries: ICCONIC hip fracture persona

**DOI:** 10.1111/1475-6773.13739

**Published:** 2021-08-14

**Authors:** Irene Papanicolas, Jose F. Figueroa, Andrew J. Schoenfeld, Kristen Riley, Olukorede Abiona, Mina Arvin, Femke Atsma, Enrique Bernal‐Delgado, Nicholas Bowden, Carl Rudolf Blankart, Sarah Deeny, Francisco Estupiñán‐Romero, Robin Gauld, Philip Haywood, Nils Janlov, Hannah Knight, Luca Lorenzoni, Alberto Marino, Zeynep Or, Anne Penneau, Kosta Shatrov, Mai Stafford, Onno van de Galien, Kees van Gool, Walter Wodchis, Ashish K. Jha

**Affiliations:** ^1^ Department of Health Policy London School of Economics London UK; ^2^ Department of Health Policy and Management Harvard T.H. Chan School of Public Health Boston Massachusetts USA; ^3^ Department of Orthopedic Surgery Brigham and Women's Hospital Boston Massachusetts USA; ^4^ Centre for Health Economics Research and Evaluation (CHERE) University of Technology Sydney Australia; ^5^ Scientific Center for Quality of Healthcare Radboud University Medical Center, Radboud Institute for Health Sciences Nijmegen The Netherlands; ^6^ Institute for Health Sciences in Aragon (IACS) Zaragoza Spain; ^7^ Dunedin School of Medicine University of Otago Dunedin New Zealand; ^8^ KPM Center for Public Management University of Bern Bern Switzerland; ^9^ Hamburg Center for Health Economics Universität Hamburg Hamburg Germany; ^10^ The Health Foundation London UK; ^11^ Otago Business School University of Otago Dunedin New Zealand; ^12^ The Swedish Agency for Health and Care Services Analysis Stockholm Sweden; ^13^ Health Division Organisation for Economic Co‐operation and Development (OECD) Paris France; ^14^ Institute for Research and Documentation in Health Economics (IRDES) Paris France; ^15^ Zilveren Kruis Leusden The Netherlands; ^16^ Institute of Health Policy Management & Evaluation University of Toronto Toronto Canada; ^17^ Brown School of Public Health Providence Rhode Island USA

**Keywords:** health systems, hip fracture, international comparisons

## Abstract

**Objective:**

This study explores differences in spending and utilization of health care services for an older person with frailty before and after a hip fracture.

**Data Sources:**

We used individual‐level patient data from five care settings.

**Study Design:**

We compared utilization and spending of an older person aged older than 65 years for 365 days before and after a hip fracture across 11 countries and five domains of care as follows: acute hospital care, primary care, outpatient specialty care, post–acute rehabilitative care, and outpatient drugs. Utilization and spending were age and sex standardized..

**Data Collection/Extraction Methods:**

The data were compiled by the International Collaborative on Costs, Outcomes, and Needs in Care (ICCONIC) across 11 countries as follows: Australia, Canada, England, France, Germany, the Netherlands, New Zealand, Spain, Sweden, Switzerland, and the United States.

**Principal Findings:**

The sample ranged from 1859 patients in Spain to 42,849 in France. Mean age ranged from 81.2 in Switzerland to 84.7 in Australia. The majority of patients across countries were female. Relative to other countries, the United States had the lowest inpatient length of stay (11.3), but the highest number of days were spent in post–acute care rehab (100.7) and, on average, had more visits to specialist providers (6.8 per year) than primary care providers (4.0 per year). Across almost all sectors, the United States spent more per person than other countries per unit ($13,622 per hospitalization, $233 per primary care visit, $386 per MD specialist visit). Patients also had high expenditures in the year prior to the hip fracture, mostly concentrated in the inpatient setting.

**Conclusion:**

Across 11 high‐income countries, there is substantial variation in health care spending and utilization for an older person with frailty, both before and after a hip fracture. The United States is the most expensive country due to high prices and above average utilization of post–acute rehab care.

**What is known on this topic**

- Health systems spend different amounts caring for patients.
- Older persons with frailty are more likely to incur high levels of spending as compared to other older populations.
- International comparisons of health systems mostly focus on the inpatient setting

**What this study adds**

- This study compares health care utilization and spending across 11 high‐income countries for an older adult with frailty across five domains of care, including acute hospital care, primary care, outpatient specialty care, post–acute rehabilitative care, and outpatient drugs.
- The United States is the most expensive country due to high prices and above average utilization of post–acute rehab care.
- Across 11 high‐income countries, there is substantial variation in health care spending and utilization for an older person with frailty, both prior to and after a hip fracture.

## 1 INTRODUCTION

A key challenge faced by many health systems is how to best design services to provide care to a small number of high‐need high‐cost (HNHC) patients. One important group of HNHC patients is older adults with frailty. Frail older adults are weak, often have multiple complex medical needs, and often require assistance for daily activities (such as dressing, eating, toileting, mobility, etc.). Frailty is a strong predictor of poor clinical outcomes.^1^, ^2^, ^3^, ^4^ In addition, the frail population is much more likely to incur high levels of spending as compared to other older populations, including higher levels of potentially modifiable spending related to avoidable hospitalizations.^5^, ^6^, ^7^ As the world population ages, and we see trends of increased longevity in older people, the incidence of frailty is expected to rise. Therefore, it is critical for health systems to identify ways to optimize care their care. One way to do this is by examining how care patterns for older patients with frailty vary across systems and, importantly, understanding how best practices can be applied from one health system to another.

A reliable marker of frailty among older adults is hip fracture,^8^ which accounts for the majority of fractures related to fragility globally.^9^ By 2050, the annual incidence of hip fracture worldwide is expected to rise over 6 million.^10^ Hip fracture is also highly associated with physical and mental disability, high mortality, and increased costs, thus requiring considerable health care resources from different parts of the health system.^11^, ^12^, ^13^, ^14^ As hip fractures almost always require a hospital admission and usually require surgery, the vast majority will be recorded in hospital admissions data and can thus serve as a robust and reliable tracer condition to explore differences in resource use across health systems.^8^

As part of the International Collaborative on Costs, Outcomes and Needs in Care (ICCONIC), we explored cross‐national variations in care trajectories and resource use for frail elders across health systems in 11 countries, which have different models of health care provision and reimbursement as follows: Australia, Canada, England, France, Germany, the Netherlands, New Zealand, Spain, Sweden, Switzerland, and the United States. We made use of hip fracture in patients older than age 65 years as a tracer condition for frailty in order to identify a comparable set of patients across the 11 health systems. Making use of patient‐level datasets linked across multiple care settings—spanning primary care, specialty services, acute hospital care, and post–acute care—we explored the variations in utilization and costs of health services across care settings and health systems in the 365 days before and after a hip fracture. Our study focuses on the following three questions: (1) how do patterns of spending and utilization of care for hip fracture patients differ across care settings in health systems that are structured and financed differently; (2) how do patterns of spending and utilization of care for these patients differ from patterns of spending and utilization in the 365 days prior to the hip fracture by country; and finally (3) to what extent do we observe notable differences in the total amount of spending and utilization of care for hip fracture patients across health systems?

## 2 DATA AND METHODS

Our methodological approach to examine variations in health systems utilization and spending combines two existing approaches that are relatively novel for international comparison of health systems. First, we proposed to use linked patient‐level data to examine the entire care pathway, rather than focusing only on care in the hospital setting. Second, our unit of analysis is a specific type of HNHC patient, which we termed a *patient persona*, whom we followed throughout the system to record instances of utilization and associated spending over the course of a year. This approach builds on the use of clinical vignette methodologies that have been used by other projects to examine resource use in the inpatient setting^15^ and by international organizations to examine variations in clinical practice.^16^

### 2.1 Data

We use linked patient‐level data from 11 countries as follows: Australia, Canada, England, France, Germany, the Netherlands, New Zealand, Spain, Sweden, Switzerland, and the United States, accessed by members of the ICCONIC collaborative. Datasets included linked data across different domains of care, including primary care, outpatient specialty care, acute hospital care, post–acute rehabilitative care, outpatient pharmaceuticals, home health care, and long‐term care. Specific details of each dataset used can be found in Table 1 of Appendix S1. Countries ability to collect comprehensive data across each domain for health care utilization and spending categories varied (Table 2 of Appendix S1).

The representativeness of the population for each dataset is found in Table 2 of Appendix S1. Data in three countries—New Zealand, Sweden, and Switzerland—covered their entire population. Data in three other countries were from specific regions—Australia (New South Wales), Canada (Ontario), and Spain (Aragon). Data in the remaining five countries were large, regionally diverse samples, including in England, France, Germany, the Netherlands, and the United States. The proportion of patients covered in each dataset varied across countries, from 3% in Spain (Aragon) and 7% in England to 100% in New Zealand, Sweden, and Switzerland. Data from most countries were from 2016 to 2017, except for Spain, Sweden, and Switzerland, which is from 2015 to 2016. Australia (2012–2016) and England (2014–2017) used data for a longer time period to allow for more observations given the size of the sample.

### 2.2 Sample selection

Using the framework from the National Academy of Medicine report “Effective Care for High‐Need Patients” as a starting point, we selected a patient persona that is representative of a frail older person. A frail older person was one of five priority populations identified as being among the most expensive to care for, have substantial health care needs, and are particularly vulnerable to poor‐quality care.^17^ The other priority populations were a person with a progressing, advanced illness; a person with complex multimorbidity; a young person with a major disability; and children with complex needs.

Our starting point was to define comparable group of patients to make up the hip persona for identification. We focused on patients older than 65 years across all systems, who were admitted to hospital with a primary diagnosis of hip fracture, which can be identified using the International Classification of Diseases–10th revision (ICD‐10) diagnostic codes, as defined by the World Health Organization: S72.0, S72.1, and S72.2. These three diagnostic codes all represent fractures in the upper part of the femur, although each code represents a different type of fracture, which may require different procedures to treat. As our group of analysis, we focus on the patients with this diagnosis who received one of three procedures: total hip replacement, partial hip replacement, or osteosynthesis (or pinning), which we identified with the relevant procedure codes in each country. The data from Spain and the Netherlands were not coded using ICD‐10 codes. Spain relied on ICD‐9 codes, and the Netherlands used comparable diagnostic codes available in the insurer data used for this study with help from clinical experts in the country. To advise on the selection of national codes and final group for analysis, we consulted with an international advisory board composed of national and international advisors from clinical, health policy, and research backgrounds (Table 3 of Appendix S1).

**FIGURE 1:**
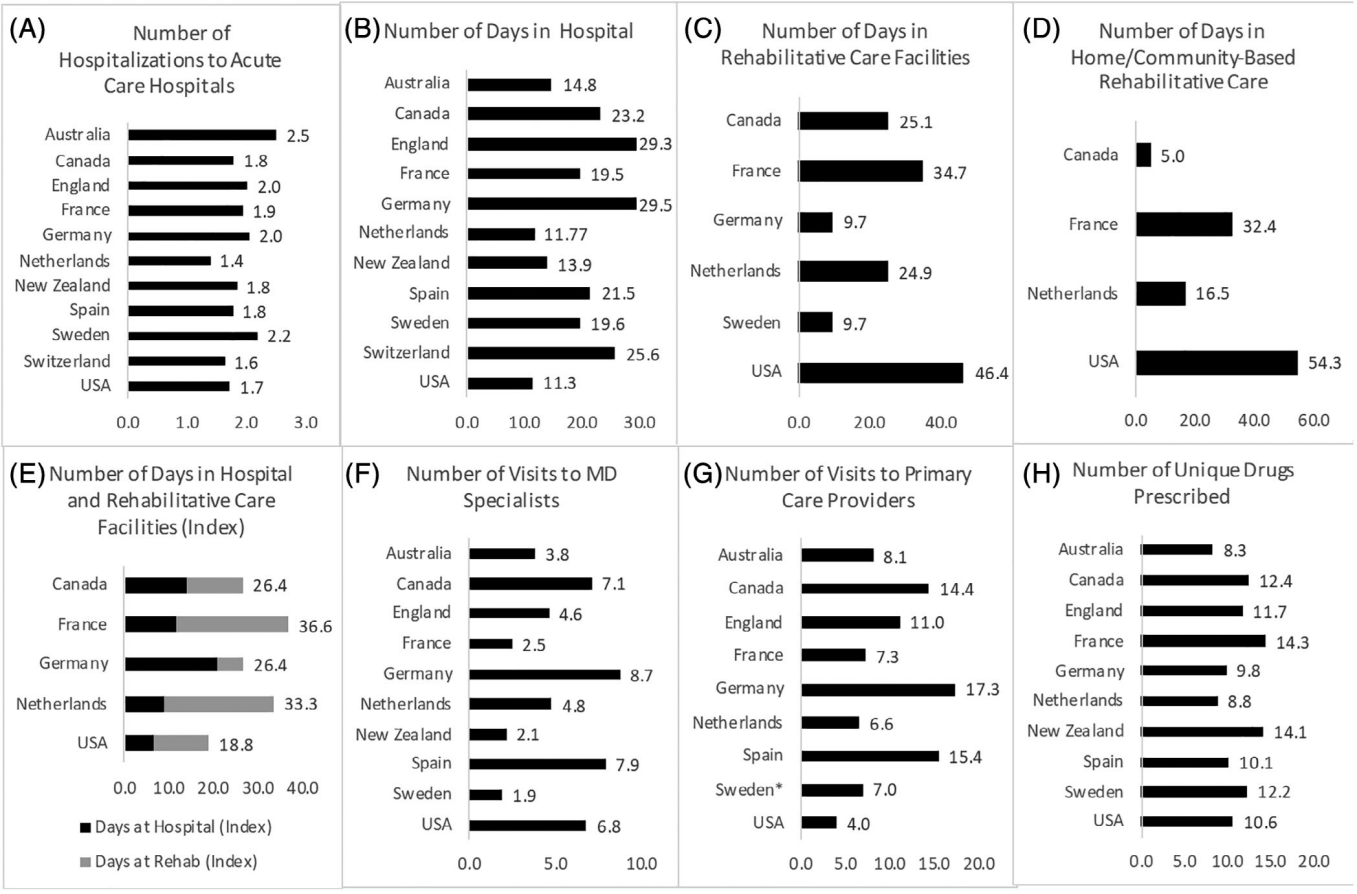
Utilization across key care settings over 365 days. *Note*: *Primary care visits for Sweden represent average yearly consumption for this cohort rather than linked patient‐level data

**FIGURE 2:**
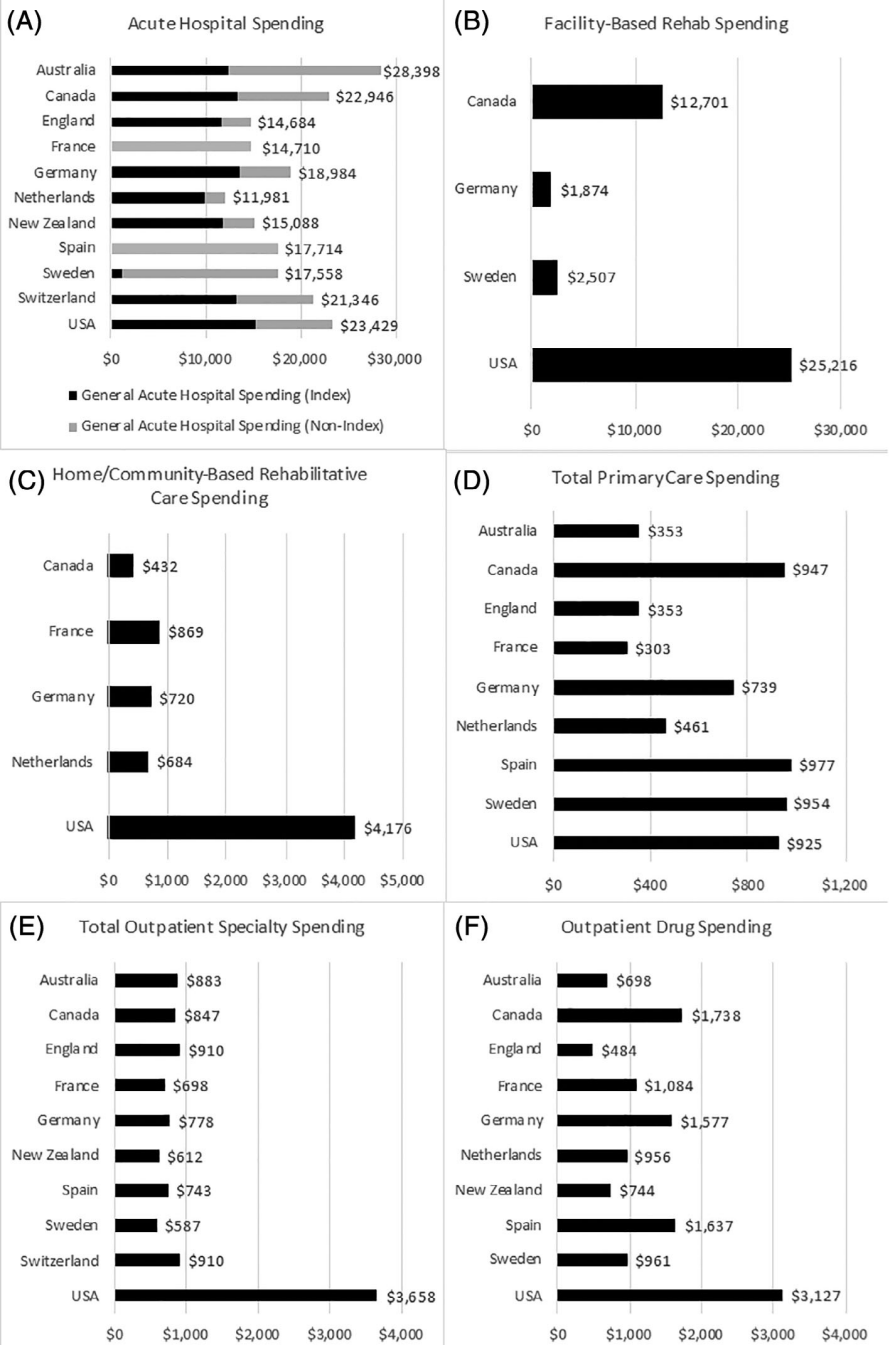
Spending across key care settings over 365 days (USD). *Note*: All figures are shown in Intl. USD. For acute hospital spending, index spending is part of the total non‐index spending. For France and Spain, the breakdown for index spending is not available; therefore, only the total spending is shown (for both index and nonindex)

**FIGURE 3:**
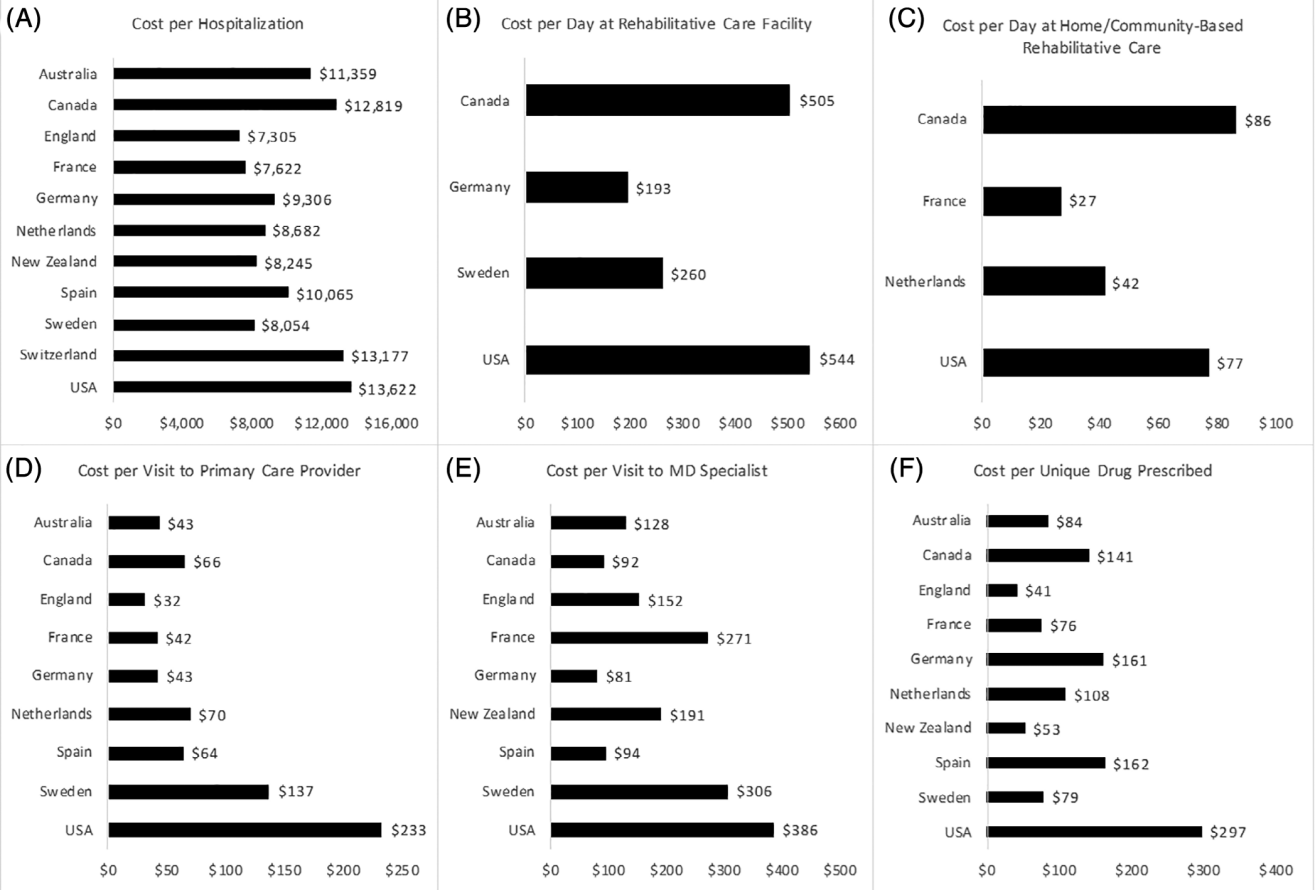
Utilization adjusted spending across key care settings over 365 days. *Note*: All figures are shown in Intl. USD. Panel A: General acute hospital spending/total hospitalizations. Panel B: Facility‐based rehab spending/total days in rehab facilities. Panel C: Home‐ or community‐based rehab spending/number of days in home‐ or community‐based rehab. Panel D: Total primary care spending/total visits to the primary care provider. Panel E: MD outpatient specialty spending/number of visits to the MD specialist. Panel E: Total outpatient pharmaceutical spending/number of unique drugs prescribed

Across countries, we tracked spending and utilization across five domains of care as follows: (1) acute hospital care, (2) post–acute rehabilitative care, (3) primary care, (4) outpatient/ambulatory specialty care, and (5) outpatient pharmaceuticals. For an outline of spending and utilization categories, please see Figures 1 and 2 of Appendix S1. To identify and follow the hip fracture persona across their pathway of care over a period of a year and establish the excess utilization and spending associated with the hip fracture, we required 3 years of patient‐level data. One year of data were used to identify all relevant hip fracture patients using the characteristics outlined above. The follow‐up year was used to measure the service use and spending incurred by each patient, across all care settings, from day 1 of hospitalization for the 365 days that follow. A look‐back year was used to establish a look‐back period of 365 days for comparison and to establish baseline utilization and spending prior to hip fracture (Figure 3 of Appendix S1). We used the look‐back year a “baseline” year across all patients and countries. Given that all spending data is age‐ and sex‐adjusted, we inferred that any additional dollar of spending observed in the year following the index hospitalization compared to the look‐back year is largely attributable to the hip fracture event per se and related complications.

### 2.3 Analysis

Due to constraints in data sharing, each country was only able to provide aggregated data for comparison. For each of the utilization and spending categories, countries supplied aggregated data reflecting mean use and spending in seven age groups (65–69, 70–74, 75–79, 80–84, 85–89, 90–94, and 95 years and up) stratified by sex.

While all countries provided expenditure data, it is important to note that we used the perspective of the health care payer across all countries. In most countries, this is carried out either directly by an insurance or sickness fund (Germany and the Netherlands) or directly from a national form of health insurance (the United States with Medicare program, Canada, etc.). Therefore, our study does not capture full costs (as it does not account for the fixed costs of all structures within a health system). It only captures the prices actually paid for the services, which across all countries, already included the fixed costs of the system. In addition, cost accounting methods used to estimate expenditure differ across countries, in part due to the differences in existing payment systems (Table 4 of Appendix S1). For example, some countries are able to report direct spending from incurred costs (those that rely on FFS entirely), while others provide information on reimbursement for specific episodes (e.g., diagnosis‐related group [DRG]) or an unweighted average unit prices. There are also differences in payment systems within countries across the different sectors. For example, pharmaceutical spending across countries reflects the amount of pharmaceutical spending in the outpatient setting and includes different amounts of out‐of‐pocket contributions. In the United States, this expenditure category captures Part D spending; in Australia and Sweden, these estimates include co‐payments. Finally, the reporting and imputation of capital investments or indirect costs also vary across system.

In order to reliably compare spending, we first applied the Organization for Economic Co‐operation and Development's (OECD) Actual Individual Consumption Purchasing Power Parities (AIC PPPs) to the expenditure data. AIC PPPs, rather than GDP PPPs, are currently used by the OECD as the most reliable economy‐wide conversion rates for health expenditure. Across each country, we applied 2017 AIC PPPs to all expenditures by age groups across the seven age groups, stratified by sex.

We then performed an age and sex direct standardization using the US sample population as the reference population for all countries. For each age group and sex, all utilization and spending measures were weighted and recalculated against the US sample population weights. The totals are then calculated by weighting each individual group and sex's shares on the original country‐specific total to generate total, male, and female age–sex standardized values. Across each category of spending and utilization, we then compared age–sex standardized results.

The institutional review board at the Harvard T.H. Chan School of Public Health approved this study.

## 3 RESULTS

### 3.1 Sample characteristics

Across the 11 countries, we identified a number of patients hospitalized with hip fracture undergoing one of the three procedures as follows (total replacement, partial replacement, or osteosynthesis): 2511 patients in Australia (New South Wales); 9872 patients in Canada (Ontario); 2738 patients in England; 42,849 patients in France; 13,998 patients in Germany; 4463 patients in the Netherlands; 2940 patients in New Zealand; 1859 patients in Spain (Aragon); 14,764 patients in Sweden; 6860 patients in Switzerland; and 29,134 patients in the United States (Table 1). The mean patient age ranged from 81.2 years (standard deviation [SD] 6.9) in Switzerland to 85.4 years (SD 7.0) in Spain. The sample was predominantly female, with the proportion of women as high as 77.1% in France and the lowest at 62.8% in Australia. Countries varied in the ability to capture secondary diagnoses in the index hospitalization, ranging from an average of 3.7 comorbidities in the United States to 1.1 in New Zealand and Canada.^18^, ^19^

**TABLE 1:** Sample characteristics

	Sweden	England	Spain	New Zealand	France	Germany	Australia	Canada	USA	The Netherlands	Switzerland
Total no.	14,764	2738	1859	2940	42,849	13,998	2511	9872	29,134	4463	6860
Mean age (SD)	83.24 (7.64)	83.49 (7.87)	85.38 (7)	83.99 (7.83)	84.26 (7.65)	83.5 (7.7)	84.67 (7.68)	83.4 (8.1)	83.2 (8.3)	82.23 (8.02)	81.24 (6.85)
% Female	67.63%	70.96%	76.71%	70.41%	77.08%	75.93%	62.80%	70.65%	71.36%	70.76%	73.72%
No. of comorbidities	1.96	2.23	3.12	1.06	2.48	3.17	2.89	1.09	3.7	0.29	2.1
Comorbidities (% of sample)											
Diabetes	14.2%	15.1%	20.2%	15.0%	13.9%	19.8%	17.6%	19.5%	22.5%	–	14.2%
CHF	11.0%	10.9%	8.1%	4.7%	15.3%	21.6%	12.4%	6.0%	17.30%	–	8.4%
Depression	2.0%	6.9%	8.3%	0.2%	9.4%	11.0%	3.9%	1.5%	15.3%	–	8.4%
Hypertension	2.0%	6.9%	8.3%	0.2%	9.4%	11.0%	3.9%	1.5%	15.3%	–	8.4%
Renal failure	5.0%	15.2%	9.0%	8.3%	9.9%	26.9%	12.2%	4.0%	19.2%	–	19.4%
COPD	9.5%	22.0%	8.0%	4.0%	7.3%	10.1%	7.3%	6.3%	22.2%	–	8.1%
S72.0 (% of total)	7908 (53.56%)	1865 (68.12%)	789 (42.44%)	1665 (56.63%)	26,657 (62.21%)	6926 (49.48%)	1411 (56.19%)	4953 (50.17%)	14,548 (49.93%)	–	3324 (48.45%)
Total replacement (% of S72.0)	1414 (17.88%)	220 (11.80%)	56 (7.10%)	297 (17.84%)	5988 (22.46%)	1558 (22.49%)	243 (17.22%)	1138 (22.98%)	1492 (10.26%)	–	1001 (30.11%)
Partial replacement (% of S72.0)	3832 (48.46%)	1184 (63.49%)	626 (79.34%)	879 (52.79%)	12,326 (46.24%)	4424 (62.88%)	704 (49.89%)	2860 (57.74%)	9245 (63.55%)	–	1934 (58.18%)
Pinning (% of S72.0)	2662 (33.66%)	461 (24.72%)	107 (13.56%)	489 (29.37%)	8343 (31.30%)	944 (13.63%)	464 (32.88%)	955 (19.28%)	3811 (26.20%)	–	389 (11.70%)
S72.1 (% of total)	5710 (38.68%)	762 (27.83%)	843 (45.35%)	1125 (38.27%)	13,543 (31.61%)	6045 (43.18%)	973 (38.75%)	4381 (44.38%)	13,274 (45.56%)	–	3147 (45.87%)
Total replacement (% of S72.1)	33 (0.58%)	*	0 (0.00%)	15 (1.33%)	396 (2.92%)	88 (1.46%)	14 (1.44%)	95 (2.17%)	114 (0.86%)	–	64 (2.03%)
Partial replacement (% of S72.1)	31 (0.54%)	*	10 (1.19%)	12 (1.07%)	213 (1.57%)	86 (1.42%)	14 (1.44%)	97 (2.21%)	317 (2.39%)	–	68 (2.16%)
Pinning (% of S72.1)	5646 (98.88%)	706 (92.65%)	833 (98.81%)	1098 (97.60%)	12,934 (95.50%)	5871 (97.12%)	945 (97.12%)	4189 (95.62%)	12,843 (96.75%)	–	3015 (95.81%)
S72.2 (% of total)	1146 (7.76%)	111 (4.05%)	227 (12.21%)	150 (5.10%)	2649 (6.18%)	1027 (7.34%)	127 (5.06%)	538 (5.45%)	1312 (4.50%)	–	389 (5.67%0
Total replacement (% of S72.2)	11 (0.96%)	*	0 (0.00%)	*	66 (2.49%)	15 (1.46%)	*	13 (2.42%)	13 (0.99%)	–	6 (1.54%)
Partial replacement (% of S72.2)	7 (0.61%)	*	14 (6.17%)	*	29 (1.09%)	4 (0.39%)	*	14 (2.60%)	26 (1.98%)	–	20 (5.14%)
Pinning (% of S72.2)	1128 (98.43%)	108 (97.30%)	213 (93.83%)	147 (98.00%)	2554 (96.41%)	1008 (98.15%)	125 (98.43%)	511 (94.98%)	1273 (97.03%)	–	363 (93.32%)
Procedure breakdown											
Total replacement (% of proc)	1458 (9.88%)	228 (8.33%)	56 (3.01%)	312 (10.61%)	6450 (15.05%)	1661 (11.87%)	258 (10.27%)	1246 (12.62%)	1619 (5.56%)	276 (6.18%)	1071 (15.61%)
Partial replacement (% of proc)	3870 (26.21%)	1235 (45.11%)	650 (34.97%)	894 (30.41%)	12,568 (29.33%)	4514 (32.25%)	719 (28.63%)	2971 (30.10%)	9588 (32.91%)	1732 (38.81%)	2022 (29.48%)
Pinning (% of proc)	9436 (63.91%)	1275 (46.57%)	1153 (62.02%)	1731 (58.88%)	23,831 (55.62%)	7823 (55.89%)	1534 (61.09%)	5655 (57.28%)	17,927 (61.53%)	2455 (55.01%)	3767 (54.91%)

*Note*: Asterisks are suppressed cells due to cell size. Hyphens are missing cells.

Abbreviations: CHF, congestive heart failure; COPD, chronic obstructive polmunary disease; SD, standard deviation.

In all countries but Spain, the most common diagnostic code was S72.0: ranging from 68.1% of the sample in England to 42.4% of the sample in Spain. The majority of patients with a hip fracture (all diagnosis) underwent osteosynthesis (pinning); for all countries apart from England, this amounted to 46.6% of the sample. Sweden had the most pinning of all countries, where 63.9% of the sample underwent this procedure. Total replacement was the least common procedure, performed on up to 15.6% of the sample in Switzerland and on as few as 3.0% of patients in Spain (Table 1).

### 3.2 Utilization differences across countries

The differences in utilization across the countries for key care settings are illustrated in Figure 1. Most patients across countries had around two hospitalizations over the 365‐day study period, including the index hospitalization (Figure 1A). Germany and England had the longest length of stay, at 29.5 and 29.3 days, respectively, nearly three times the average length of stay in the United States (11.3) and the Netherlands (11.7) (Figure 1B). Of the countries that had data on the facility‐based rehabilitative care sector, Sweden (9.7) had the fewest number of days spent in this sector, while the United States (46.4) had the highest number (Figure 1C). When combined, France and the United States had the highest number of days in hospitals and rehab facilities over the year, summing to a total of with 54.2 days per person in France and 57.6 days per person in the United States. Of the four countries that had data on utilization of home‐ and community‐based rehab care, the United States also had the most (54.3 days), again followed by France (32.4 days). Canada and the Netherlands had far less at 5 days and 16.5 days, respectively (Figure 1D). Of the total days spent in hospital and institutional rehab, for most countries, the majority was concentrated in the index admission, with the exception of the United States (Figure 1E).

The number of unique visits to primary care providers and outpatient specialists varied considerably across countries (Figure 1F,G). On average, the United States had the fewest number of visits to primary care providers (4.0) over the course of the year, while Germany (17.3) had the most. When combined, Germany and Spain had the highest number of visits across both specialists and primary care providers. The United States was the only country to have more MD specialist visits than primary care doctor visits.

France had the greatest number of unique drugs prescribed at 14.3 drugs per person. While Australia had the fewest unique drugs prescribed (8.3 drugs per person) (Figure 1H). The United States was an average utilizer with 10.6 drugs per person.

### 3.3 Spending differences across countries

The differences in spending across different care settings are illustrated in Figure 2, while Figure 3 shows differences in spending taking into account differences in utilization. There was a large difference in total spending for acute hospital care, ranging from $28,398 per person in Australia to $11,981 in the Netherlands (Figure 2). The index hospitalization accounted for the majority of the spending in this category for most countries (see Figure 2A). When accounting for the number of hospitalizations over the year, the United States had the most spending per hospitalization ($13,622), closely followed by Switzerland ($13,177), and England had the lowest cost per hospitalization ($7305) (Figure 3A).

Of the countries that were able to measure facility‐based rehabilitation spending, the United States spent the most per patient over the course of the year ($25,216), nearly double than the next highest spender Canada ($12,701) (Figure 2B). There was a similar pattern for total expenditure of home‐ and community‐based rehab spending, where the United States spent $4176 per patient per year, compared to $869 in France (the next highest spender) and $432 in Canada (the lowest spender) (Figure 2C). While the United States had the greatest number of institutional and home‐ and community‐based rehabilitation days, they also had the most expensive institutional rehab per day ($544) when compared to others, followed closely by Canada ($505 per rehabilitation day) (Figure 3B). Canada had the most expensive home‐ and community‐based rehabilitation day ($86 per day) followed by the United States ($77) (Figure 3C).

There were differences across the countries in spending related to primary care services, with Spain, Canada, Sweden, and the United States all spending around $900 per year and Australia, England, and France all spending closer to $350 per year (Figure 2D). Most of the difference in total seems to be accounted for by the number of visits, with most countries spending between $32 and $70 per visit. The exception is the United States and Sweden who spent $233 and $137 per visit, respectively (Figure 3D).

There was little variation across countries related to total outpatient specialty care spending over the year with most countries spending around $800 per person, apart from the United States that spent $3658 per person (Figure 2E). However, the cost per MD specialist visit was more varied, with the United States spending comparatively more (Figure 3E). Outpatient drug spending was the highest in the United States ($3127 per person) and the lowest in England ($484 per person) (Figure 2F). When adjusting for drug utilization, England had the lowest cost per drug prescribed ($41) followed by New Zealand ($53); the United States had the highest cost per drug ($297) (Figure 3F).

### 3.4 Comparison to the look‐back year

**FIGURE 4:**
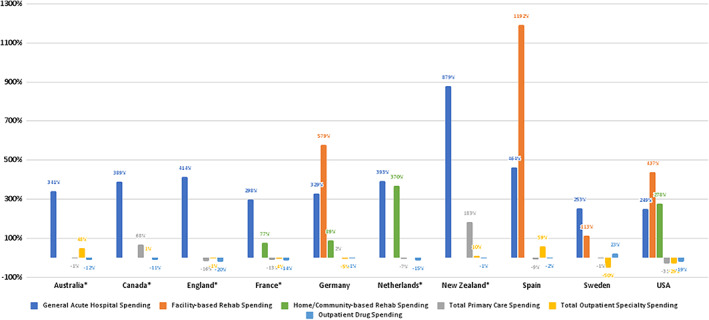
Percent change in expenditure (look‐back year compared with current year). *Note*: Percentage values are calculated as (current year expenditure – look‐back year expenditure)/(look‐back year expenditure) for each care setting [Color figure can be viewed at wileyonlinelibrary.com]

Using the expenditure in the 365 days prior to the hip fracture as baseline expenditure for this persona, we examined the relative increase in expenditure for each care category in the period 365 days following the expenditure. All countries saw a large increases in the general acute care spending in the year following the hip fracture relative to the 365 days before. This was most pronounced in the countries that had the lowest baseline expenditure such as New Zealand, where expenditure for this care setting increased by 879% (from $1541), as compared to 253% in Sweden (from $4972) and 437% in the United States (from $5868) (Figure 4, Appendix 5). Of the countries that were able to collect rehab data, whether facility based or in the home/community, all countries saw an increase in spending relative to the look‐back year. The greatest increase in facility‐based rehab was in Germany, who had very low baseline spending. The United States saw a large increase in rehab spending (437%) despite having already high baseline spending in this care setting, averaging at $4693 per patient. The Netherlands and the United States saw the greatest increase in home‐ and community‐based rehab (370% and 278%, respectively), although the United States was spending nearly four times as much in the baseline year ($3864 per patient compared to $1123 per patient) (Figure 5 of Appendix S1).

Spending in the other three categories (primary care, outpatient specialty, and outpatient drugs) did not uniformly increase across countries, with many experiencing a decline in the expenditures associated with these care settings instead. In the study year, England, France, the Netherlands, and the United States, all saw small decreases in relative expenditures across all three of these categories as compared to the look‐back year. Canada (68%) and Germany (2%) saw increases in primary care spending of different degrees, while Australia (48%) and Spain (59%) saw increases in relative outpatient specialty expenditure.

## 4 DISCUSSION

In this study, we examined patterns of utilization and spending for an older frail adult recovering from a hip fracture across 11 countries as follows: Australia, Canada, England, France, Germany, the Netherlands, New Zealand, Spain, Sweden, Switzerland, and the United States. The resources used by this HNHC persona vary considerably across countries, although the United States stands out as the highest spender in every care setting. Across all countries, considerable resource use is devoted to inpatient care and rehabilitative care, both in facilities and in the community. We found that this group of patients also have high expenditures in the year prior to the hip fracture, mostly concentrated in the inpatient setting and also make greater use of primary care and outpatient specialty care relative to the general population as measured by the OECD.^20^ This is likely related to underlying frailty but also varies across countries, suggesting that differences in how health services are organized and provided may also play a role. Our study demonstrates the importance of looking across the system to understand the true resource use of complex patients, which is essential if policy makers want to identify areas for improvements in care.

Our results have important implications for policy makers interested in better understanding the relative performance of their country with regards to care for this population. For example, our findings suggest that the United States appears to be the least efficient in caring for this persona, in both price and quantity. Across all sectors, the United States spends more per unit of health care use in addition to having more and longer durations of time spent in facility‐based rehab and home rehab than other countries. In addition, when we sum days spent in acute and post–acute care settings over the course of the year, the United States becomes the highest utilizer of care overall. These patterns may be culminating a number of different issues in the care pathway. It is possible that patients are discharged “quicker but sicker” from the hospital in the United States because of the widely accessible post–acute care infrastructure covered by Medicare.^21^, ^22^ Other countries, like England, do not have a comprehensive provision of accessible post–acute care service and, instead, observe much longer hospital length of stay.^23^ Another possibility is that US patients have less access to affordable long‐term care, as it is not covered by the Medicare program.^24^ This may lead to a substitution of care, where long‐term care services are being provided in the post–acute setting (predominately skilled nursing facilities).

Among the countries able to provide information on post–acute rehab utilization and costs, we observed that those with universal long‐term care systems (such as Canada, the Netherlands, and Sweden) spend fewer days and subsequent lower costs in the post–acute rehab setting. This again suggests that in countries without easily affordable and accessible long‐term care, care and costs are being shifted into the post–acute setting. In the case of England, which has neither comprehensive long‐term care nor post–acute care coverage, we observed that the hospital setting bears a bigger burden relative to other countries in terms of longer length of stay. Our findings have important implications for those concerned with allocative efficiency of health care spending. Particularly for this patient group, the setting where care happens can have substantial repercussions on total spending. Countries like the Netherlands and Sweden are structured to utilize more long‐term care than other costly alternatives.

Another key objective of this work was to quantify the resource use of a frail older patient and how it changes when they suffer an acute event. The look‐back year reveals to us that, even prior to the hip fracture, frail older patients have high levels of health care utilization and costs. An acute event such as hip fracture causes costs in these settings to balloon, largely driven by increases in inpatient and rehabilitative care. Interestingly, many countries see expenditures fall across the primary care and outpatient specialty settings likely because they are institutionalized or become deceased.

Our work also sheds light on other factors that may contribute to the higher US expenditure for this persona. In the outpatient setting, we found important differences in the relative distribution of primary care and specialty care services. The United States is the only country that utilizes more speciality care visits than primary care, both in the year before and after the hip fracture. Despite being an average utilizer of unique drugs per person, the United States spends substantially more. On the other hand, England and New Zealand stand out as the lowest per unit spenders, which may be explained by well‐documented differences in the various economic and regulatory instruments used. For example, in England, the National Institute of Health and Care Excellence regulates the use and price of new drugs.

New Zealand is one of the few countries that have an effective tendering system for pharmaceuticals, which may contribute to lower prices.^25^, ^26^

This work makes important contributions to the literature on international comparisons. While several European projects have demonstrated the differences that exist across European health systems in the intensity of care delivered,^27^ the basket of services delivered,^15^ treatment pathways,^28^ outcomes,^29^ and costs^15^, ^28^, ^30^ for patients with similar diagnoses, most have focused on comparisons of the inpatient setting. To our knowledge, only one other project, EuroHOPE, has compared patient trajectories over time, although for a different subset of countries, patients, and years. Our study builds upon this work by looking specifically at HNHC patients and considering chronic disease, rather than only acute, care using contemporary data. Survey work in these countries has also demonstrated differences in the coordination of care for HNHC patients, highlighting particular challenges for primary care providers specifically.^31^

Our work has some key limitations. First, though we have done our best to ensure data comparability across countries, there are some differences in the types of data used as well as in the representativeness and completeness of data across countries, which may influence the comparability of estimates across certain categories. In the United States specifically, our sample of data represents patients covered by the Medicare fee‐for‐service system, which may incur different spending to those covered through Medicare advantage. Second, there are differences in national coding practices and cost accounting practices of the data across countries that in turn may influence the results, for example, countries with fee‐for‐service systems will have more precise estimates of expenditure than those with global budgets where costs have been estimated using a top‐down approach. We have tried to document these as much as possible so as to identify potential sources of bias. In addition, the expenditure estimates reported only reflect the costs incurred by the payer and, for the most part, do not capture any additional out‐of‐pocket payments, which are likely to vary across countries and care settings. The notable exception to this is for outpatient pharmaceutical payments where some countries do capture co‐pays, such as Australia and Sweden. We reported differences in coverage across countries alongside our results, so that readers can better understand where these types of payments are likely to exist by country. Third, while all results are age and sex adjusted, we do not adjust for comorbidities, which may influence expenditure and utilization. For example, the United States has higher comorbidities than most of the other countries, which may contribute to higher spending. Finally, while we aimed to compare the entire care pathway across countries, most countries are missing some utilization or expenditure data from key care settings particularly around post‐acute and long‐term care. This limits the extent we are able to compare total spending and utilization or better understand patterns of substitution between care settings. However, we believe that the linked data we have currently collected are among the most comprehensive data reflecting the care pathway across countries.

## 5 CONCLUSIONS

Most cross‐country comparisons to date have focused on looking at variations in the utilization and cost of hospital care.^27^, ^28^, ^29^ Our results illustrate that across health systems, there is considerable variability with regards to the relative share of care, and expenditures, that occurs in hospitals for this patient group. Limiting comparisons to only the inpatient setting likely provides a misleading picture of resource use for these patients. Until we have a broader perspective of the distribution of resources across the care pathway, health policy will remain fragmented and miss out on the biggest opportunities to improve care for this group of patients and improve the efficiency of health care systems.

## DISCLAIMERS

The results from New Zealand are not official statistics. They have been created for research purposes from the Integrated Data Infrastructure (IDI), which is carefully managed by Stats NZ. For more information, about the IDI please visit https://www.stats.govt.nz/integrated-data/.

The 45 and Up survey data used to represent Australia oversamples people above of age 80 years and residents of rural and remote areas (45 and Up Study Collaborators; 2008). The 45 and Up Study had a response rate of 18%, so the cohort might not be representative of the NSW population. Also, the survey focuses on NSW and may not be representative of the national sample for the same age group.

## Supporting information


**Appendix S1** Supporting information
