# Supplementary material for: Differences in health care spending and utilization among older frail adults in high‐income countries: ICCONIC hip fracture persona
Source: Health Serv Res. 2021 Aug 14;56(Suppl 3):1335–46. doi: 10.1111/1475-6773.13739 (PMC8579209; doi:10.1111/1475-6773.13739)

**Appendix Table of Contents:**

1. **Appendix Table 1.** Country Dataset Information
2. **Appendix Table 2:** Representativeness of Country Datasets
3. **Appendix Table 3**: Available Data by Country
4. **Appendix Table 3.** ICCONIC Advisory Board Members
5. **Appendix Table 4.** Health System Characteristics by Country
6. **Appendix Figure 1:** Identification Strategy for Hip Fracture
7. **Appendix Figure 2:** Diagnosis and Procedure code breakdown by country
8. **Appendix Figure 3.** Identifying spending across distinct components of care across the health system
9. **Appendix Figure 4.** Defining categories of utilization across distinct care domains
10. **Appendix Figure 5:** Lookback Year Spending Across Care Settings
11. **Appendix Figure 6:** Lookback Year Utilisation Across Care Settings

**Appendix Table 1.** Country Dataset Information

| **Country** | **Datasets** |
| --- | --- |
| Australia | - Sax Institute’s 45 and Up study |
| Canada | - Administrative claims data of the province of Ontario from the Ontario Ministry of Health and the Canadian Institute for Health Information through the Institute for Clinical Evaluative Sciences (ICES) |
| England | - Primary care data from the Clinical Practice Research Datalink (CPRD) linked to secondary care data from Hospital Episode Statistics (HES) and Office for National Statistics (ONS) death register |
| France | - SNDS (Système National des Données de Santé/National Health Data System) - ResidEhpad (long-term care in residential facilities) |
| Germany | - Administrative data of a large, nationally active health insurance with more than 8m enrollees (BARMER) (includes utilization/costs of all sectors that are paid by health insurance) |
| Netherlands | - Zilveren Kruis insurance data (nationwide), which has about 30% of market share in the country |
| New Zealand | - The Integrated Data Infrastructure (IDI) - The National Minimum Dataset (NMDS) (hospital admissions data) - The pharmaceutical collection (medication dispensing data) - The National Non-Admitted Patient Collection (NNPAC) (outpatient data) |
| Spain | - Base de datos de usuario (National Health Service users dataset including insurees admin data) - OMI-AP (primary care electronic health records) - Conjunto Mínimo Básico de Datos (CMBD) (admin data for hospital discharges and outpatient contacts) - Sistema de Información Hospitalaria (outpatient visits to specialized care) - Receta Electrónica (e-Prescription files) - Facturación Recetas (billing files of over-the-counter prescriptions) - Puesto Clínico Hospitalario de Urgencias (emergency care contacts) |
| Sweden | - The national patient registry (inpatient and outpatient specialized care) - The national prescription drug registry (outpatient pharmaceuticals) - The national mortality registry - The national registry for interventions in municipal healthcare (enrollment in home medical care) - The national registry of measures for the elderly and people with disabilities (long term care) - Regional administrative registers of primary care consumption for the regions of Stockholm, Jönköping, Norrbotten, Skåne and Västra Götaland. |
| Switzerland | - Medical statistics dataset of the Federal Statistical Office (FSO), including hospital admissions records - Patient data from hospital-based outpatient care dataset of the FSO - Short and long-term care facility records dataset of the FSO |
| United States | - Medicare fee-for-service (FFS) data, 20% sample of all patients age 65 years or older (Part A, B and D) |

**Appendix Table 2.** Representativeness of Country Datasets

|  | **Australia** | **Canada** | **England** | **France** | **Germany** | **Netherlands** | **New Zealand** | **Spain** | **Sweden** | **Switzerland** | **USA** |
| --- | --- | --- | --- | --- | --- | --- | --- | --- | --- | --- | --- |
| **Year of Data** | 2012 to 2016 | 2016 to 2017 | 2014 to 2017 | 2016 to 2017 | 2016 to 2017 | 2016 to 2017 | 2016 to 2017 | 2015 to 2016 | 2015 to 2016 | 2015 to 2016 | 2016 to 2017 |
|  | | | | | | | | | | | |
| **Country Population** |  |  |  |  |  |  |  |  |  |  |  |
| Total | 23.5 million | 35.2 million (Canada 2016) | 55.3 million (in 2016) | 64.5 million | 82.2 million | 16.9 million | 4.7 million | 46.6 million | 9.9 million | 8.4 million | 323.1 million |
| Population age 65+ | 3.5 million | 5.9 million (Canada 2016) | 9.9 million  (in 2016) | 12.3 million | 16.8 million | 2.4 million | 733,272 | 8.3 million | 1.9 million | 1.5 million | 49.2 million |
|  | | | | | | | | | | | |
| **Sample Population (Dataset)** |  |  |  |  |  |  |  |  |  |  |  |
| Representativeness of dataset | Regional data (New South Wales) | Regional Data (Ontario Province) | National sample | 12 regions of France | National sample | National sample | Full national data | Regional Data (Aragon, ES) | Full national data | Full national data | National sample |
| Total sample in dataset | 267,086 | 14.5 million | 7% of the UK population, representative in terms of age and sex* | 43.1 million | 8.6 million | 30% market share | 4.7 million | 1.3 million | 9.9 million | 8.4 million | 6.4 million |
| Population age 65+ in dataset | 123,625 | 2.5 million |  | 8.2 million | 2.5 million | 30% market share | 733,272 | 288,738 (21.73%) | 1.9 million | 1.5 million | 5.4 million |

Note: This table shows the sample coverage for each country’s sample dataset. Specific full denominator was not able to be shared by data supplier in the Netherlands.

**Appendix Table 3.** Available Data by Country

|  | **US** | **CA** | **DE** | **FR** | **ES** | **UK** | **AU** | **NZ** | **NL** | **CH** | **SE** |
| --- | --- | --- | --- | --- | --- | --- | --- | --- | --- | --- | --- |
| **Utilization** | | | | | | | | | | | |
| **Inpatient/Acute Care** | | | | | | | | | | | |
| General Acute Hospitalizations | X | X | X | X | X | X | X | X | X | X | X |
| Days in Hospital | X | X | X | X | X | X | X | X | X | X | X |
| Days in Hospital (Index) | X | X | X | X |  | X | X | X | X | X | X |
| **Post-Acute Care Rehab** | | | | | | | | | | | |
| Days in Rehabilitative Care Facility | X | X | X | X |  |  |  |  | X |  | X |
| Days in Rehabilitative Care Facility (Index) | X | X | X | X |  |  |  |  | X |  |  |
| Home or Community-Based Rehab Days | X | X |  | X |  |  |  |  | X |  |  |
| **Primary Care** | | | | | | | | | | | |
| Total Primary Care Visits | X | X | X | X | X | X | X |  | X |  | X |
| Primary Care MD Visits | X | X | X | X | X | X | X |  | X |  | X |
| Primary Care Visits to Nurses or Equivalent | X | X |  |  | X | X | X |  | X |  | X |
| **Outpatient Specialty Care** | | | | | | | | | | | |
| MD Specialist Visits | X | X | X | X | X | X | X | X | X |  | X |
| **Drugs** | | | | | | | | | | | |
| Number of Unique Drugs Prescribed | X | X | X | X | X | X | X | X | X |  | X |
| **Spending** | | | | | | | | | | | |
| **Inpatient/Acute Care** | | | | | | | | | | | |
| Total Inpatient/Acute Hospital Spending  (General and Psychiatric) | X | X | X |  | X | X | X | X | X | X | X |
| Total General Acute Hospital Spending | X | X | X | X | X | X |  | X | X | X |  |
| Total Psychiatric Spending | X | X | X |  |  | X |  |  | X |  |  |
| General Acute Hospital Spending (Index) | X | X | X |  |  | X | X | X | X | X | X |
| **Post-Acute Care Rehab** | | | | | | | | | | | |
| Facility-Based Rehab Care Spending | X | X | X |  |  |  |  |  |  |  | X |
| Facility-Based Rehab Care Spending (Index) | X | X | X |  |  |  |  |  |  |  |  |
| Home-Based Rehab Care Spending | X | X | X | X |  |  |  |  | X |  |  |
| **Primary Care** | | | | | | | | | | | |
| Total Primary Care Spending (MD and Non-MD) | X | X | X | X | X | X | X |  | X |  | X |
| Primary Care MD Services Spending | X | X | X | X | X | X | X |  | X |  | X |
| Primary Care Non-MD Services Spending | X | X |  |  | X | X | X |  | X |  | X |
| **Outpatient Specialty Care** | | | | | | | | | | | |
| Total Outpatient Specialty Care Spending  (MD Specialty and Other) | X | X | X | X | X | X | X | X |  | X |  |
| Outpatient MD Specialty Services Spending | X | X | X | X | X | X | X | X |  | X | X |
| **Drugs** | | | | | | | | | | | |
| Outpatient Drug Spending | X | X | X | X | X | X | X | X | X |  | X |
| **Day of Procedure (hip persona only)** | | | | | | | | | | | |
|  | X | X | X | X | X | X |  |  |  |  | X |

Notes: This table shows data availability across countries and health care settings for utilisation and spending for the hip persona. An “X” for each cell marks that data is available and provided in the ICCONIC data collection. An empty cell denotes that the category could not be provided.

Appendix Figures 3 and 4 further define these categories.
Country legend: US = United States, CA = Canada, DE = Germany, FR = France, ES = Spain, UK = England, AU = Australia, NZ = New Zealand, NL = Netherlands, CH = Switzerland, SE = Sweden.

**Appendix Table 4.** ICCONIC Advisory Board Members

| **Country** | **Member** | **Title** |
| --- | --- | --- |
| **Meeting Chairs** | Peter C. Smith, MSc | *Emeritus Professor of Health Policy, Imperial College London* |
|  | Melinda K. Abrams, MS | *Senior Vice President, Delivery System Reform and International Innovations, The Commonwealth Fund* |
|  | Andrew Street | *Professor of Health Economics, London School of Economics and Political Science* |
| **Australia** | Philip Haywood | *Senior Research Fellow, Centre for Health Economics Research and Evaluation* |
|  | Sallie Pearson | *Professor, UNSW Centre for Big Data in Health Research* |
|  | Jason Thompson | *Unit Head \| Economics, Expenditure & Medicare Unit \| Australian Institute of Health and Welfare* |
| **Canada** | Rhona McGlasson, MBA, PT | *Executive Director, Bone and Joint Canada* |
|  | Fredrika Scarth, PhD, MA | *Director, Secretariat, Premier’s Council on Improving Healthcare and Ending Hallway Medicine, Ontario Ministry of Health* |
| **England** | Peter C. Smith, MSc | *Emeritus Professor of Health Policy, Imperial College London* |
|  | Antony Johansen, MBBS | *Consultant Orthogeriatrician, University Hospital of Wales*  *Clinical Lead, National Hip Fracture Database, Royal College of Physicians* |
| **France** | Sandrine Colas, PhD | *Pharmacoepidemiologist, Real World Insights* |
|  | Antoine Rachas, MD, PhD | *Public Health Doctor, French National Health Insurance (CNAM)* |
| **Germany** | Reinhard Busse, Dr. Med., MPH | *Professor and Head of the Department of Health Care Management, Berlin University of Technology* |
|  | Jens Deerberg-Wittram, MD | *CEO, RoMed Kliniken* |
| **Netherlands** | Patrick Jeurissen, PhD, MPA | *Professor, Radboud University Medical School*  *Science Officer, Ministry of Health, Welfare and Sports* |
| **New Zealand** | Richard Hamblin | *Director for Health Quality Intelligence at our Health Quality and Safety Commission* |
|  | Lisa Gestro | *Executive Director of Primary and Community Strategy, Southern District Health Board* |
| **Spain** | Ismael Said, MD, MSc | *Specialist in Internal Medicine, Hospital Universitario Ramón y Cajal* |
|  | Francisco Estupiñán-Romero, MD | *Researcher, Health Science Institute in Aragón (IACS)* |
|  | Carlos Martín Hernández, MD, PhD, MSc | *Specialist, Orthopedic Surgery and Traumatology*  *Associate Professor of Orthopaedic Surgery and Traumatology, Zaragoza University* |
| **Sweden** | Jean-Luc af Geijerstam, MD, PhD | *Executive Director, The Swedish Agency for Health and Care Services Analysis* |
|  | Cecilia Rogmark, MD, PhD | *Orthopaedic Surgeon, Skane University Hospital*  *Associate Professor, Lund University* |
| **Switzerland** | Lars Clarfeld, Dr. med. | *General Secretary, Swiss Society of General Internal Medicine (SGAIM)* |
| **United States** | Eric Schneider, MD, MSc | *Senior Vice President for Policy and Research, The Commonwealth Fund* |
|  | Andrew Schoenfeld, MD, MSc | *Orthopedic Surgeon, Brigham and Women’s Hospital*  *Associate Professor of Orthopedic Surgery, Harvard Medical School* |

**Appendix Table 5.** Health System Characteristics by Country^32,33^

| **Health System Characteristics** | **Australia** | **Canada** | **England** | **France** | **Germany** | **Netherlands** | **New Zealand** | **Spain** | **Sweden** | **Switzerland** | **United States** |
| --- | --- | --- | --- | --- | --- | --- | --- | --- | --- | --- | --- |
| Health system expenditure per capita (Intl. $ 2019) | $5,187 | $5,418 | $4,653 | $5,376 | $6,646 | $5,765 | $4,204 | $3,616 | $5,782 | $7,732 | $11,072 |
| Long term care expenditure per capita (Intl. $ 2017) | $101 | $923 | $726 | $780 | $1,099 | $1,385 | - | $317 | $1,425 | $1,409 | $512 |
| Health system expenditure % of GDP (2019) | 9.3% | 10.8% | 10.3% | 11.2% | 11.7% | 10.0% | 9.3% | 9.0% | 10.9% | 12.1% | 17.0% |
| Long term care expenditure % of GDP (2017) | 0.2% | 1.9% | 1.8% | 1.8% | 2.1% | 2.6% | - | 0.9% | 2.9% | 2.4% | 0.9% |
| Type of system (NHS type, private insurance, social insurance) | National public health insurance | National public insurance | National health care system (NHS) | Statutory insurance through employment-based funds, tax-financed coverage for unemployed | Mostly statutory insurance with some private insurance | Statutory, mandatory insurance through 11 private non-profit carriers | National health care system | National health care system | National health care system with decentralized service delivery | National health insurance for basic coverage with optional supplementary insurance plans | Mix of public and private insurance |
| Population coverage (%) | 100% | 100% | 100% | 100% | 100% | 100% | 100% | 100% | 100% | 100% | 91.5% |
| Payment system of hospitals | Public hospitals mostly activity-based (DRG) payments, with the rest global budgets while private hospitals mainly FFS | Global budgets, some case-based payment | Mostly case-based payments, with some local bundled-payment pilots | Mostly case-based (DRG) payments | Case-based (DRG) payments | Case-based (DRG) payments with a global budget | Case-based payments | Mostly global budgets, some episode-based payments | Mostly global budgets, remainder case-based (DRG) payments or PFP | Case-based (DRG) payments for inpatient care, FFS for outpatient care | Mix of FFS, case-based (DRG), and per-diem payments |
| Payment system of primary care (FFS, capitation, P4P, hybrid) | Mostly FFS, some PFP | Mostly FFS, some alternative payments or salaries | Mix of capitation, FFS, PFP | Mix of FFS and PFP, capitated annual bonus for chronic diseases | FFS | Mix of capitation and FFS, some bundled payments and PFP | Capitation and FFS, some incentive payments | Global budgets, capitation, PFP | Mostly capitation, some FFS or PFP | Mostly FFS, some capitation | Mostly FFS, some capitation and incentive payments |

**Appendix Figure 1.** Identifying spending across distinct components of care across the health system


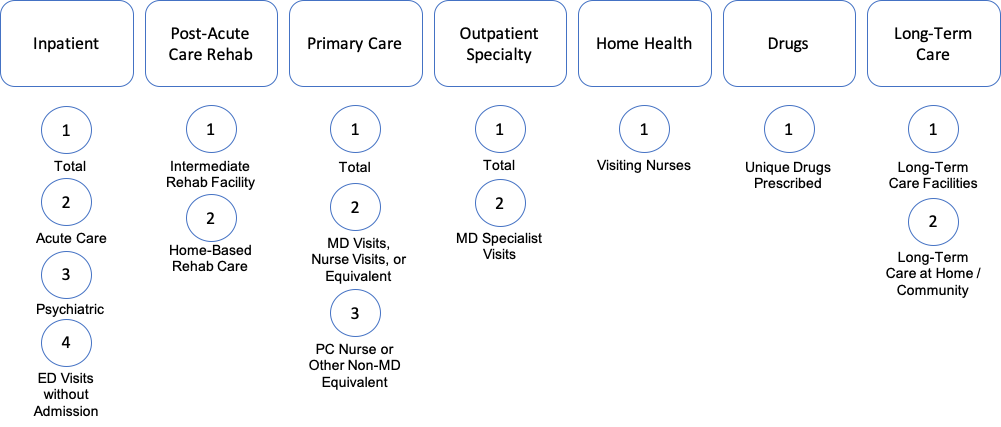


Notes: This figure details each component of expenditure measured across countries. Total indicates a sum of all subcategories indicated below.

**Appendix Figure 2.** Defining categories of utilization across distinct care domains


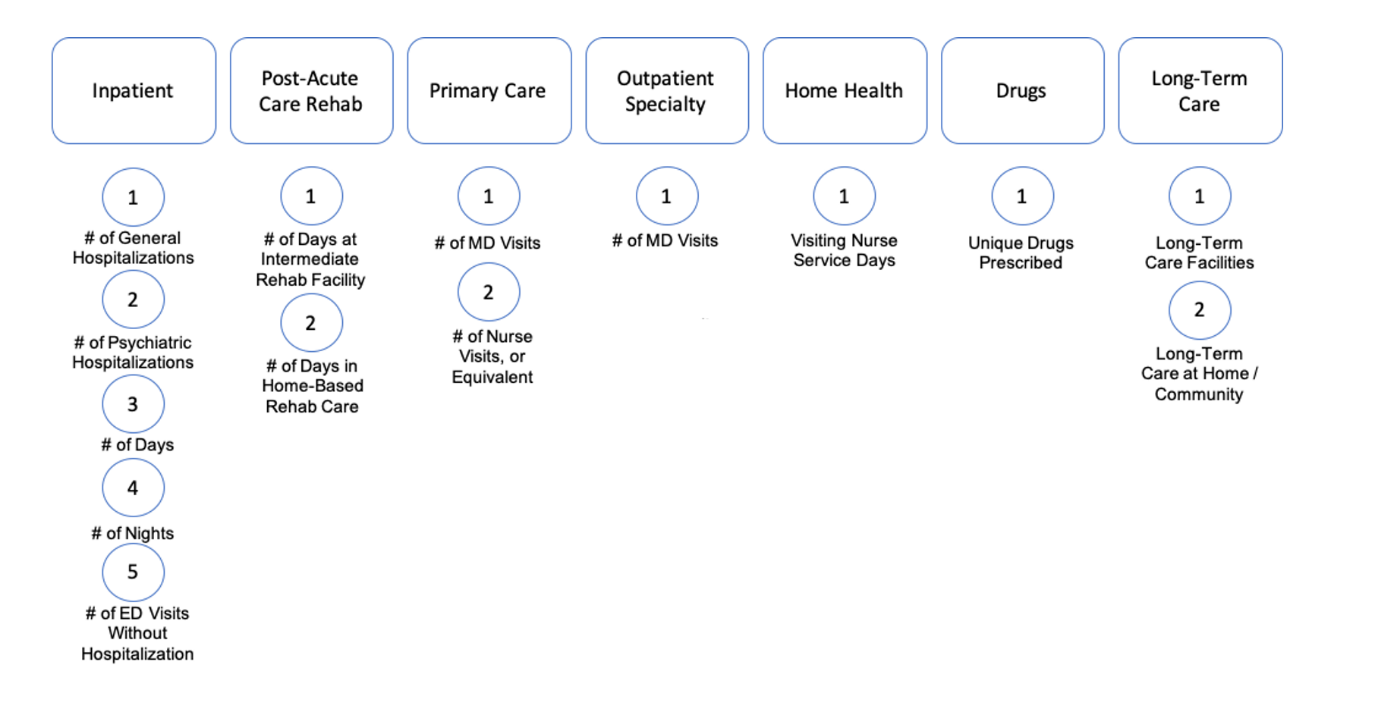


Notes: This figure details each component of utilization measured across countries.

**Appendix Figure 1:** Identification Strategy for Hip Fracture

**
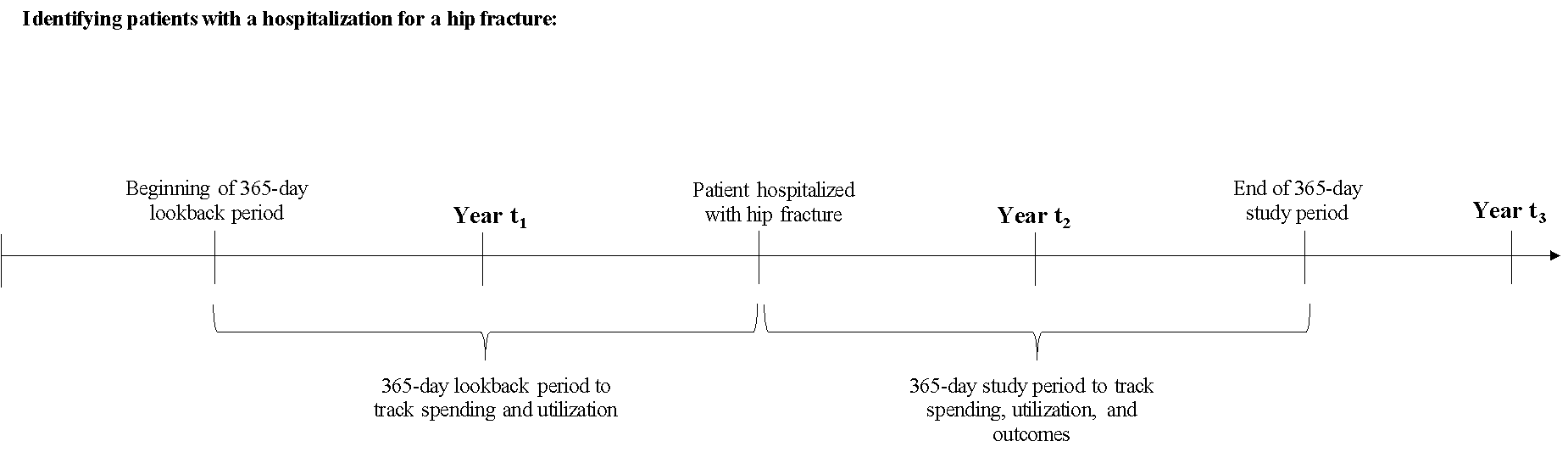
**

**Appendix Figure 4: Lookback Year Spending Across Care Settings**


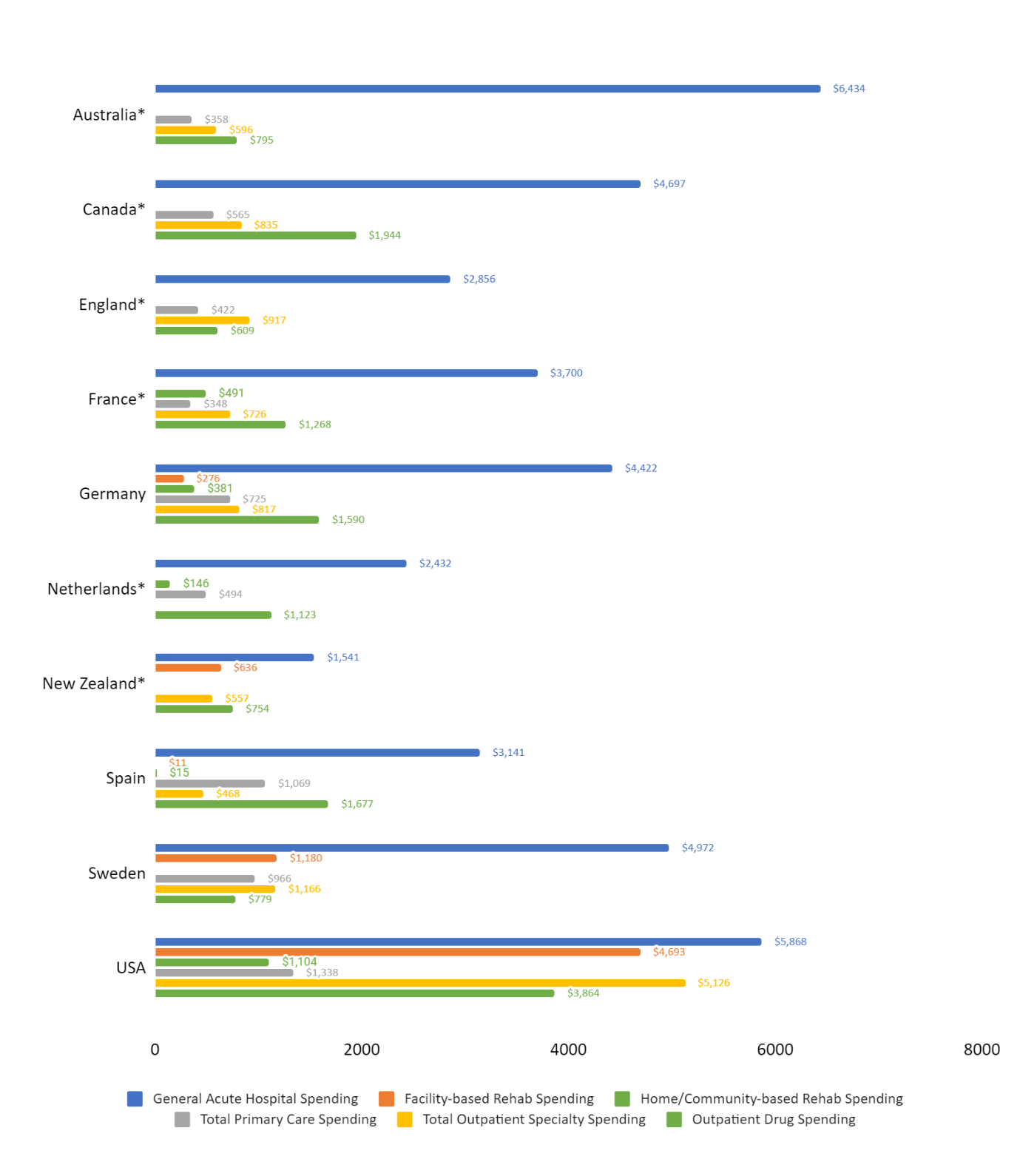


Note: All figures are shown in Intl. USD.

**Appendix Figure 5: Lookback Year Utilisation Across Care Settings**


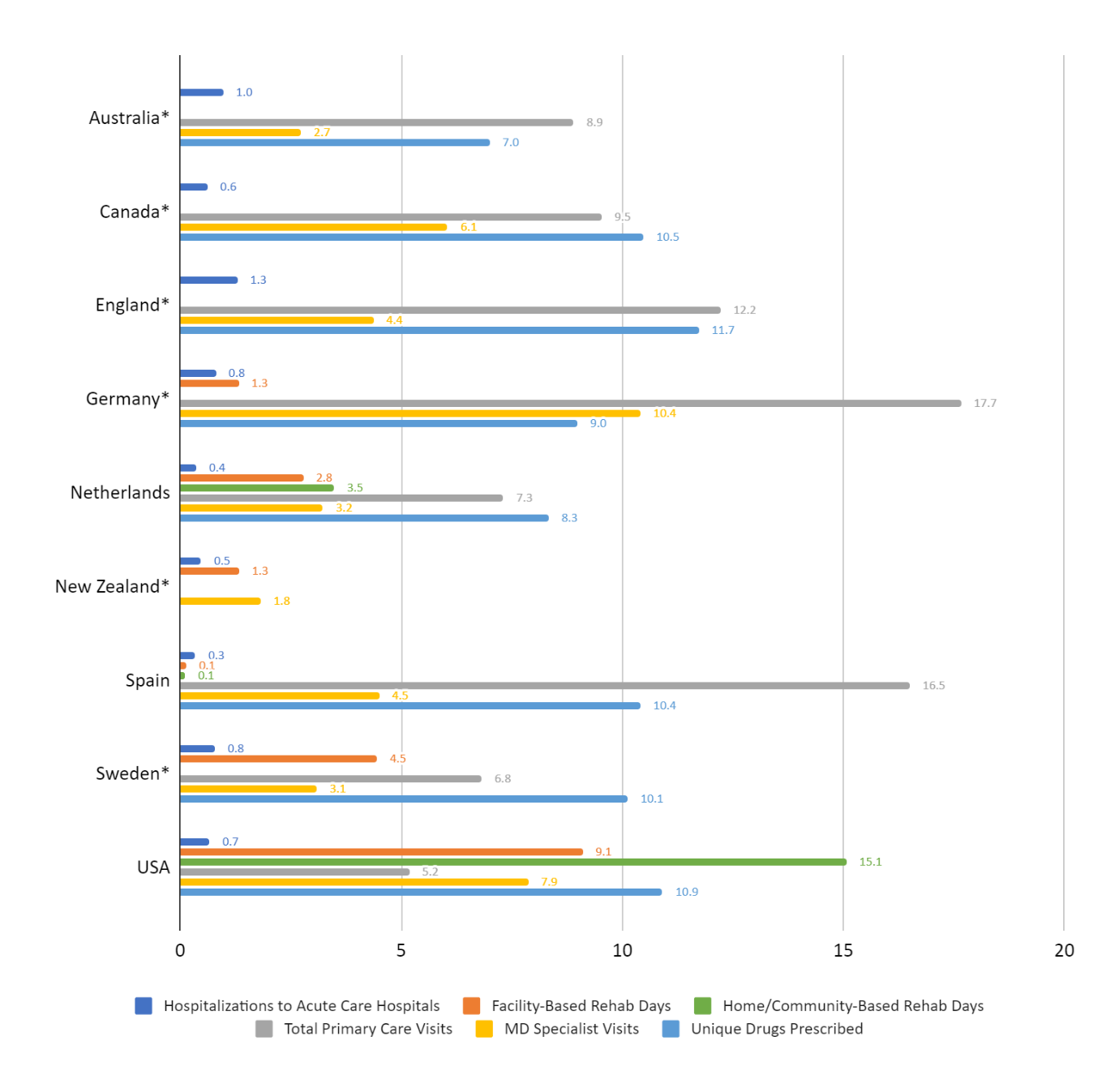

Supplement: Supplementary file 1 — Appendix S1 Supporting information [file HESR-56-1335-s001.docx]
